# COVID-19 pandemic and financial market volatility: A quantile regression approach

**DOI:** 10.1016/j.heliyon.2023.e21131

**Published:** 2023-10-17

**Authors:** Sabeeh Ullah, Sumaira Khan, Nazia Iqbal Hashmi, Md Shabbir Alam

**Affiliations:** a Institute of Business and Management Sciences (IBMS), Faculty of Management & Computer Sciences (FMCS), The University of Agriculture, Peshawar, Pakistan; b Department of Management Sciences, Women University, Swabi, Pakistan; c Department of Finance, College of Business Administration, Prince Sultan University, Riyadh, Saudi Arabia; d Department of Economics and Finance, College of Business Administration, University of Bahrain, Sakhir, Bahrain

**Keywords:** COVID-19 pandemic, Volatility, Quantile regression, Financial markets

## Abstract

The study examined the nexus between the COVID-19 pandemic and the market volatility of the global markets. For this purpose, a 30-country sample was used based on the most COVID-19 cases and deaths during the study period, from January 1 to December 12, 2020. We employed panel quantile regression and Panel Estimated Generalized Least Square (Panel-EGLS) frameworks to analyze the influence of COVID-19 on volatility in the whole sample and subsamples of emerging and developed markets. Our results of Panel-EGLS showed that the new cases and deaths positively impact volatility in the naïve and control models. The results from quantile regression also illustrated that new deaths and cases have positively influenced market volatility at the 50th and 75th quantiles. From the subsamples, our results demonstrate almost similar signs and significance for the impact of COVID-19 on market volatility in developed and emerging markets in both the naïve and control models. Both the results illustrate that any increase in COVID-19 positively caused volatility in the whole and subsamples at the mean and upper quantile levels. Our results necessitate coordinated global government actions to stabilize markets, mitigate volatility's impact by proactive policies in future health crises, and underscore a monetary policy for stability.

## 1 Introduction

The coronavirus (hereafter COVID-19) originated in Wuhan, China, in late 2019 and spread to almost all parts of the world, reaching a global scale by early 2020. COVID-19 is a health crisis, a humanitarian disaster [1], and a fast-spreading disease [2] caused by the SARS-CoV-2 virus [3]. On March 11, 2020, the World Health Organization (WHO) declared the COVID-19 outbreak a global pandemic [2]. As of July 6, 2023, the COVID-19 pandemic has caused more than 6 million deaths and infected more than 700 million people worldwide [4]. COVID-19 has changed people's lifestyles and brought countless challenges worldwide [5].

The COVID-19 pandemic also triggered a global economic crisis, as the government imposed a range of lockdown tools like calling off public events, closure of business activities, schools, and universities, international travel controls, and suspension of many companies, all of which had substantial implications for businesses and economies worldwide. Contemporary studies identified that the world financial markets and economy were affected by social distancing and lockdown during the COVID-19 pandemic [2,6]. Moreover, the COVID-19 pandemic has had far-reaching consequences on society, including high risk to commodities and financial markets [7]. Stock markets worldwide experienced unprecedented volatility during this period, reflecting the uncertainty and economic disruptions caused by the pandemic. As a result, investors faced heightened uncertainty and rapidly changing market conditions, leading to substantial fluctuations in stock market returns.

Understanding the relationship between the COVID-19 pandemic and stock market volatility is crucial for investors, policymakers, and researchers seeking to navigate and mitigate the impact of such crises. Many countries across the globe were in trouble because of the COVID-19 fast-spreading disease and unexpected shocks in economic trends, which suddenly changed investors' decisions due to anxiety and risk [2,8] and led to significant losses to investors [9]. Therefore, researchers recommended it is helpful to consider COVID-19 and its influences on global financial markets [10]. Similarly [2], argued that researchers have significant room to examine financial markets and investor decisions after the COVID-19 outbreak. Since the COVID-19 crises are international, they have a local impact on human lives, markets, and economies [11].

Moreover, the uncertain nature of the COVID-19 outbreak and its impact on the economy makes it difficult for policymakers to develop appropriate economic policies [12]. Likewise, the Great Depression of 1990 and the Global financial crises of 2008, COVID-19 also pushed global economies into a recession by reproducing financial volatility [13]. Additionally, during the COVID-19 pandemic, the negative influence of volatility on financial returns and multiple crashes on international investment has become a significant issue for investors around the globe [14]. Due to speculative bets in financial markets, investors generate an influx of financial transactions, creating extreme speculative bubbles and volatile behaviour in financial markets [14]. For investors and policymakers, it is essential to assess the dynamics of volatility in the markets because the exact economic impact of COVID-19 has not yet been cleared [9].

This study aims to explore financial market volatility during the COVID-19 pandemic using an advanced panel data framework. Our study addresses the literature gaps and contributes to the existing body of knowledge in several ways.

First, contemporary studies (such as [[15], [16], [17], [18]]) used the standard GARCH (1,1) procedure for measuring volatility. At the same time, the standard GARCH procedure assumes a symmetric effect on the volatility by ignoring the leverage effect on the stock [19]. Thus, our study contributes by measuring market volatility through the standard deviation of returns using the Exponential-GARCH (EGARCH) procedure (proposed by Ref. [20]), which is the most appropriate model to capture market volatility during the pandemic. Ref. [14,21] argued that EGARCH is the best model to measure market volatility during economic fluctuations. Additionally, the stock returns are highly dependent on the market volatility behaviour, which leads to the existence of asymmetry between the market volatility and returns ([14,22], which minimizes diversification's benefit [23]. Thus, empirically, volatility reacts asymmetrically to the sign of the COVID-19 shocks and, therefore, can be best covered using the exponential-GARCH volatility procedure.

Second, some studies have explored the effect of the COVID-19 pandemic on stock market volatility (see, e.g. Refs. [9,[24], [25], [26], [27], [28]],), they have often been limited to short event window period, failing to capture the long-term effect of the fast-spreading disease and its impact on interconnected financial markets. During COVID-19, the connectedness between volatility and the financial market has increased [26,29,30] because the shocks of COVID-19 have been faster and more severe on the global economy [17]. Considering a longer-period dataset of stock market indices from various countries, this research article provides insights into the worldwide impact of the COVID-19 pandemic on stock market volatility. A longer period can capture the economic and social consequences of the COVID-19 outbreak [31]. Also, contemporary research documented that the impact of COVID-19 on economies in the long term is high in terms of business failure and unemployment [[32], [33], [34]].

Third, existing studies have explored the impact of the pandemic on stock market volatility; they have often been limited to specific countries or regions, failing to capture the global nature of the crisis and its impact on interconnected financial markets. Because of globalization, global markets' interdependency increases, increasing the interdependence of stock markets [35]. Ref. [36] argued that one country's crises spread rapidly to other countries. Likewise, Ref. [37] reported that one country's return shocks were transmitted to other countries due to connectedness. Due to this interdependency, the study tries to investigate the impact of COVID-19 on market volatility for both severely affected developed and developing countries through an advanced panel data framework.

Fourth, existing studies have often focused on the mean effects of the pandemic on stock market volatility, assuming that the relationship is uniform across the entire return distribution (see, for example, [5,14,37,38]). However, this assumption may not hold during extreme uncertainty and market stress, such as the COVID-19 pandemic. Ref. [39] found extreme asymmetric volatility during the pandemic negatively correlated with the stock return. Quantile regression enables researchers to examine how the pandemic affects stock market volatility differently at various points in the distribution, thereby providing a more nuanced understanding of the relationship. Therefore, this study innovated the combination of Panel-EGLS and Quantile regression methods, which has reference to accounts for correcting endogenous repressors' biases, sensitivity to outliers, mitigates non-Gaussian error problems, and potential asymmetries in panel data.

To fill the literature gaps, the main motives of this research study is to contribute to the existing body of knowledge by analyzing the relationship between the COVID-19 pandemic and stock market volatility by providing a comprehensive analysis that accounts for potential asymmetries, extreme events, and global market interdependencies, offering valuable insights for investors, policymakers, and researchers. As per the available literature, the study's novelty is that it is the first to investigate the nexus between COVID-19 and financial market volatility in emerging and developed markets.

The remainder of the paper is arranged as follows: Section 2 illustrates the background literature and development of the hypothesis. Section 3 provides the methodological design. Section 4 reports the empirical findings, and section 5 gives the conclusion and policy recommendations of the study.

## 2 Literature review

Prior research has examined the nexus between pandemics and market volatility, providing valuable insights that the pandemics can have on stock markets during global health crises. For instance, during the SARS epidemic 2003 [40], documented a significant decline in market indices and increased market volatility in affected countries. Likewise, Ref. [41] reported increased market volatility during the SARS epidemic in highly infected countries. Moreover, Ref. [42] observed a similar pattern in market volatility during the H1N1 influenza outbreak in 2009, indicating the importance of considering the nexus between pandemics and the stock market. Ref. [43] examined market liquidity and returns. They reported that the market return was severely affected by the COVID-19 outbreak in the U.S. and reported a negative association between COVID-19 and market returns. The spreading of COVID-19 caused a reduction in economic activity, which led to risks to financial market stability [44]. The financial market stability in Africa and Nigeria has been hit by COVID-19 [1]. Ref. [45] examined the effect of COVID-19 on the Indonesian economy and concluded that COVID-19 caused market volatility, significantly negatively influencing Indonesian economic growth.

### 2.1 COVID-19 pandemics and market volatility

In early 2020, the emergence of the COVID-19 pandemic brought disruption and a new level of uncertainty to global markets. Numerous studies have investigated the nexus between the COVID-19 pandemic and stock market volatility, clarifying the unique challenges posed by the COVID-19 pandemic.

Research has shown that the shocks of the COVID-19 outbreak have been faster and more severe to the global economy as the market has become more volatile [17]. Researchers have also examined the consequences of COVID-19 in China and different countries. China is the first affected country whose economy was hit by COVID-19 [12]. Companies worldwide have started a contraction in production because most companies depend upon inputs from China as the central hub for FDI in Asia [2]. Global supply chain functioning has been interrupted due to limited and restricted transport among the countries, further slowing economic activity [12]. COVID-19 affects both the demand and supply sides of the global value chain of China and the U.S. trade war [46]. According to the IMF, China's growth rate fell by 0.4 % compared to 5.6 % of its initial growth rate and slowed down by 0.1% point growth globally [47]. The U.S. is the 2nd most suffered country in terms of trade loss, with a trade impact of $5779 million [48]. In March 2020, the share value of corporate companies in the U.S. fell by 20 % [49]. Ref. [50] forecast that the economic growth in the U.S. will decrease by 3.8 % and by 6.3 % in the European Union. World Bank predicted that the world health crisis 2020 is moving towards the worst global recession after World War II [51].

Further, Ref. [52] confirmed that the COVID-19 cases have adversely affected stock returns in Vietnam. They employed event study procedures before and during the lockdown and concluded that the financial sector of Vietnam was severely hit during the COVID-19 outbreak. Still, after the lockdown, Vietnam's financial sector successfully controlled the pandemic and recovered from its stock market losses. Ref. [53] examined the impact of COVID-19 on four Central European countries' markets and reported that the outbreak of COVID-19 has changed the expectations of financial market participants. They found a negatively significant relationship between COVID-19 and financial markets. Ref. [31] examined the spread of COVID-19 and its impact on the global economy. They predicted a reduction in general economic activity through a lockdown and social distancing hit the economy and stock market. Still, on the other hand, it creates an opportunity for many governments to repair their public healthcare sectors through reforms such as the focus on infrastructure and disease detection systems in hospitals.

Many governments can take the COVID-19 pandemic as an opportunity to fix the problems in the healthcare and economic systems [31]. Ref. [43] analyzed the return and liquidity of the U.K. market. They found that during the COVID-19 outbreak, the market return and liquidity have declined, which badly hit the U.K. leading indices FTSE-100. For this purpose, the Bank of England introduced a 330 billion loan (15 % of GDP) program to support businesses and decreased interest rates by 65 bases, sliding to 0.1 % [43]. Ref. [54] revisit the effect of COVID-19 on market volatility in Asian countries. They documented a reducing trend in the impact of pandemic control measures and new COVID-19 cases on market volatility since 2021 at the regional and country levels. Ref. [55] examined the effect of COVID-19 on the 30 most virus-affected countries worldwide using a panel data estimation framework and found that new COVID-19 cases negatively influenced stock market returns in developed and developing markets. However, the new daily deaths due to coronavirus positively influenced stock market returns in emerging markets. The study has only considered market return as a dependent variable, while it has been suggested that future research should consider other dependent variables (e.g., market volatility) [55].

Moreover, Ref. [56] documented that studies on the influence of COVID-19 on market volatility are scarce and quickly growing. Various factors are attributable to the impact of COVID-19 on market volatility. These factors include lockdown measures, changes in consumer behaviour, disruptions to global supply chains, and uncertainty surrounding the pandemic's public health and economic implications, creating an environment of heightened market uncertainty and amplified volatility.

Due to these factors and scarce literature, this study is motivated to analyze the impact of COVID-19 on financial market volatility. Table 1 summarizes the studies conducted on COVID-19 and the volatility nexus.

**Table 1:** Summary of empirical literature.

Authors	Sample Countries	Period	Methods	Findings
[56]	07 countries	One year	GARCH and VAR	↑ COVID-19 ↑Market Return
[24]	U·S.	Three months	Least Squares Estimation	↑ COVID-19 ↑Market volatility
[57]	09 countries	Three months	EGARCH procedure	↑ COVID-19 ↑Market volatility
[9]	15 countries	Two months	Minimum Spanning Tree Method	↑ COVID-19 ↑Market volatility
[58]	10 countries	Six months	EGARCH procedure	↑ COVID-19 ↑Market volatility
[18]	India	Ten months	GJR-GARCH Model	↑ COVID-19 ↑Market volatility
[59]	75 countries	Three months	Fixed Effect Model	↑ COVID-19 ↑Market volatility
[60]	15 countries	Six months	GARCH Model	↑ COVID-19 ↑Market volatility
[13]	Asia Pacific countries	Four months	Continuous Wavelet Transformation (CWT), GJR-GARCH	↑ COVID-19 ↓Market volatility
[61]	U.S. and China	Ten months	Wavelet analysis, Q.Q. approach	↑ COVID-19 ↑Market volatility
[28]	27 countries	Four months	Fixed effect, Driscoll and Kraay (D.K.) estimator	↑ COVID-19 ↑Market volatility
[62]	39 countries	One year	Fixed effect, Random effect	↑ COVID-19 ↑Market volatility

### 2.2 Theoretical support

The current study examined the nexus between the COVID-19 pandemic and market volatility within the framework of behavioural finance theory. According to behavioural finance theory, investors exhibit irrational behaviour in various situations due to psychological biases and emotions that deviate from rational behaviour and can influence their decision-making process [63]. During the COVID-19 pandemic, heightened levels of uncertainty, panic, and fear can lead to irrational behaviour of investors and increased stock market volatility [64]. Ref. [65] reported that fear of COVID-19 resulted in randomness in the stock market. Likewise, Ref. [66] documented that COVID-19 created fear among investors and brought market plunges and volatility spikes in the U.S. options and equity markets. Moreover, behavioural biases such as over-anchoring bias, herding behaviour, loss aversion, and reaction and under-reaction can amplify market volatility during crises [67]. This theory provides a concrete theoretical foundation for analyzing the impact of COVID-19 on market volatility.

### 2.3 Hypothesis development

#### 2.3.1 COVID-19 and market volatility

Compared to the 2008 financial crisis, COVID-19 caused financial market volatility [9]. Ref. [2] analyzed the short-term influence of COVID-19 in 21 countries, including the UK, Germany, the USA, Japan, Italy, and Singapore. They indicated that after the COVID-19 health crises, the stock markets of most affected countries fell quickly compared to other countries' stock markets in Asia and experienced more negative abnormal returns. For the U.S. market, Ref. [43] reported a negative association between COVID-19 and market returns. Similarly, Ref. [45] concluded that COVID-19 caused market volatility. Ibrahim et al. (2020) studied the relationship between COVID-19 health crises, market volatilities, and government measures in the Asia-Pacific region. They reported that the government responses to COVID-19 reduced market volatility in most sampled countries, such as Malaysia, Vietnam, and Laos, which faced medium volatility. At the same time, Japan, China, the Philippines, and South Korea experienced high volatility. The pandemic's potential economic losses and uncertainty caused financial market volatility [68]. Based on this evidence, our study hypothesizes that:

Ha: The COVID-19 pandemic positively influences financial market volatility.

#### 2.3.2 COVID-19 new cases and financial market volatility

The COVID-19 virus fast-spreading disease increases and doubles new infections cases even quicker every two to three days. The fear of the pandemic caused many companies to close completely, so financial markets have been volatile [2]. Due to high uncertainty and its linkage with economic losses, COVID-19 causes markets to become more volatile and unpredictable, which increases the global market risk [9]. A more significant number of infected people, especially in China, Italy, Japan, the U.S., Korea, and Iran, damage financial stability and reduce global economic activity [44]. The stock market continues to move in the same direction as the number of COVID-19 cases increases in different countries, economic shocks, and the cost of COVID-19 increases significantly as many countries experience COVID-19 shocks concurrently [46]. Ref. [16] suggested that COVID-19 causes market volatility and drives public attention. They further argued that a 1 % rise in COVID-19 cases resulted in a 0.8 % decrease in the market return. Based on these arguments, we can hypothesize that:

Hb: COVID-19 new cases positively influence financial market volatility.

#### 2.3.3 COVID-19 new deaths and financial market volatility

Ref. [27] investigated financial markets during the outbreak of COVID-19 and revealed that COVID-19 deaths negatively affect the market. Due to a significant increase in COVID-19 deaths, the global financial markets crashed, and several leading world indices fell in March 2020 [69]. There is some connection between the COVID-19 outbreak and economic activities [70], as an increase in COVID-19 decreases economic activities and causes financial market instability [44]. Ref. [57] documented that COVID-19 deaths in sampled countries negatively affect many financial securities' returns. Ref. [71] found that market volatility increases as confirmed deaths increase. Ref. [18] argued that the Indian market experienced volatility during the COVID-19 outbreak. Likewise, Ref. [56] documented that deaths due to COVID-19 affect the U.S. market volatility. Based on these arguments, we conclude and hypothesize as follows:

Hc: COVID-19 deaths positively affect financial market volatility.

## 3 Research design

### 3.1 Data description and sample

To examine the nexus between the COVID-19 pandemic and market volatility, panel data from January 1 to December 12, 2020, were chosen. The reasons behind selecting this period (i.e., the year 2020) are: (i) the initial surge in the COVID-19 pandemic due to deaths, economic and mental health was severely affected in the year 2020, which was becoming under control due to the development and use of efficacious vaccines in early 2021 [72]. (ii) In early 2021, the global economy experienced a positive impact due to vaccination [66].

Our sample comprises 30 highly affected countries based on new COVID-19 confirmed deaths and cases reported by the World Health Organization (WHO). The sampled countries' details and their markets are shown in Appendix A1. These countries were further divided into subsamples of developed and emerging countries based on Morgan Stanley Capital International (MSCI) world classification of countries. The study compiles data from three databases, namely Global Change Data Labs (GCDL) at Johns Hopkins University, the Center for Systems Science and Engineering (CSSE), and a global financial website (www.investing.com), after refining the data by excluding those countries whose data for daily new confirmed cases and deaths are either unavailable or constant throughout the study period. Our final dataset of 30 countries comprised 14 developed and 16 emerging countries. For definitions and data collection sources of all the variables, see Table 2.

**Table 2:** Variables definition and sources.

Variables	Symbol	Definition	Data Source
Market volatility	FMV	The standard deviation of returns was calculated using the EGARCH procedure.	investing.com
New-cases	NC	The logarithm of daily base COVID-19 confirmed new cases.	CSSE
New-deaths	ND	The logarithm of daily base COVID-19 confirmed new deaths.	CSSE
Stringency Index	S.T. Index	Government preventive measures for COVID-19 spreading [9,62]	GCDL
Extreme poverty Index	E.P. Index	People live in extreme poverty [73].	GCDL
Life expectancy Index	L.E. Index	Mean life at the time of birth in the year 2019 [74].	GCDL
Human Development Index	H.D. Index	The average key dimensions are a long and healthy life, knowledge, and good living standards [75].	GCDL

Note: At Johns Hopkins University, the Center for Systems Science and Engineering (CSSE) and Global Change Data Labs (GCDL) and.

### 3.2 Operationalization of variables

#### 3.2.1 Financial market volatility (FMV)

Financial market volatility is the dependent variable. For financial market volatility, we used the EGARCH model presented in Equation (1), a well-known and extensively used method in finance for calculating market volatility [76].

(1) $$\ln\;{\delta_{it}}^{2}=\alpha_{t}+\beta_{i}\;\ln\left(\delta_{it-1}^{2}\right)+\varphi \frac{\varepsilon_{t-1}}{\sqrt{\delta_{t-1}^{2}}}+\eta \left[\frac{\varepsilon_{t-1}}{\sqrt{\delta_{t-1}^{2}}}-\sqrt{\frac{2}{\pi }}\right]$$

Where ${\delta_{it}}^{2}$ is the conditional variance, $\alpha_{t}$ is a conditional density function, $\beta_{i}$ is the perseverance in conditional variance without the movement in the market, φ is the leveraging effect, and η is the GARCH effect.

#### 3.2.2 COVID-19 new confirmed cases and deaths

The COVID-19 new confirmed cases (NC) and deaths are the primary independent variables. Following [2,9,24,26,55], we used the logarithm of new COVID-19 infected confirmed cases and deaths reported were used as a proxy for the COVID-19 pandemic. Pandemic fear due to new cases and deaths caused many companies to close down completely, leading to increased financial market volatility [2].

#### 3.2.3 Control variables

This study used indices of Stringency, Life expectancy, Extreme poverty, and Human Development as control variables.

*Stringency Index:* Following Ref. [62], the stringency index is used as a control variable for estimating the impacts on financial market volatility. The stringency index is the level of restrictions by the government on closures of schools, workplaces, and public events, restrictions on public transport, national and international travelling, restrictions on stay-at-home requirements, and restricted commercial activities, causing the financial market to be more volatile in both emerging and developed markets [62]. Further, Ref. [62,77] documented that the closure of workplaces and schools and restrictions on national and international travelling significantly impact market volatility in emerging economies. In financial markets, the closure of public transport contributed to higher volatility [9]. Government intervention robustly and significantly increases the volatility in the international stock market [78]. By examining the government actions and their impact on market returns in 77 countries using data from January 22 to April 17, 2020, Ref. [79] documented that the government announcement of social distancing has adversely influenced market returns and economic activity and indirectly reduced new COVID-19 cases. It is concluded that the restricted policies and actions of the government in social destining brought significant economic damage [80].

*Extreme Poverty Index:* The extreme poverty index is essential to assessing the effect of COVID-19 on market volatility. Government intervention and policy action designed to minimize the risk of the virus are more important in economic activity (business activities have fallen quickly because of policy action) [73]. An extreme poverty index measures the proportion of people living below the poverty line. According to the International Labor Organization, working hours were reduced by 17 % in the first three-quarters of 2020, equal to 500 million full-time jobs lost globally [81]. The U.S., Europe, and other developed countries took monetary and fiscal measures for the compensation of income losses of workers and businesses and to control economic crises. However, this response is very limited in emerging economies [16]. With the increase in COVID-19, the GDP growth and market performance decreased due to less public attention towards businesses (buying and selling) during the COVID-19 pandemic. Further, Ref. [16] argued that the fear of COVID-19 drives public attention and causes market volatility.

*Life Expectancy Index:* The primary metric of population longevity and health is the life expectancy at birth, the average number of years a newborn would live throughout their life span to death [74]. From 2019 to 2020, in 27 out of 29 countries, the life expectancy at birth was reduced due to the increase in mortality above the age of 60. The COVID-19 deaths, especially among males in Lithuania and the USA, experienced more significant losses in life expectancy in 2020 [74]. Due to a substantial rise in COVID-19 deaths, the global market crashed, and several key world indices fell in March 2020 [69].

*Human Development Index (HDI):* A positive relationship exists between the human development index (HDI) and COVID-19 in countries like the USA, Italy, Spain, Germany, and the U.K [82]. HDI is an aggregate index of living standard, life expectancy, and education, which is positively correlated with the infection rate and fertility rate of COVID-19 and positively related to average annual salary and chronic disease [75]. A significant number of COVID-19-infected people, especially in China, Italy, Japan, the U.S., Korea, and Iran, reduced economic activity and damaged global financial stability [44]. The Stock Market continues to move in the same direction as the number of new COVID-19 cases increases in different countries, economic shocks and the cost of the COVID-19 pandemic increase significantly as many countries experience COVID-19 shocks concurrently [46].

### 3.3 Analytical model

To examine the nexus between the COVID-19 pandemic and stock market volatility, we adopt the two-step procedure of Ref. [24,83]. First, a naïve estimation is implemented for Eq. (2) without using any control variables. Second, Eq. (3) was estimated by incorporating all the control variables. The baseline models for Eqs. (2), (3) are as follows:

(2) $$Y_{it}=\alpha +\beta_{1}Y_{it-1}+\beta_{2}COVID-19_{it-1}+\varepsilon_{it}$$

(3) $$Y_{it}=\alpha +\beta_{1}Y_{it-1}+\beta_{2}COVID-19_{it-1}+\sum_{j=1}^{n}\delta_{j}contro{l_{j}}_{it}+\varepsilon_{it}$$

Where $Y_{it}$ represents market volatility; COVID-19 represents the new confirmed cases and deaths; control represents the control variables used in the study.

Additionally, the whole sample is divided into two subsamples of developed and emerging markets based on the classification of Morgan Stanley Capital International (MSCI) and re-estimate Eq. (2) and Eq. (3) for each subsample.

### 3.4 Econometric procedure

Following Ref. [[84], [85], [86], [87]], we employed two econometric approaches, namely Quantile regression and Panel-EGLS to estimate Eqs. (2), (3).

#### 3.4.1 Panel quantile regression

Prior studies on the nexus between COVID-19 and market volatility employed different econometric approaches like regression models [54], event studies [5,38], and GARCH models [14]. In such cases, Ordinary Least Squares (OLS) may generate biased findings [88]. These approaches are primarily concerned with the mean effect estimation of the dependent variables and are more sensitive to outliers and normality assumptions. In contrast, the application of panel quantile regression is relatively limited in this context. Quantile regression offers a distinct advantage over traditional regression models by allowing for the investigation of the relationship between the COVID-19 pandemic and stock market volatility across different quantiles of the stock market return distribution. This approach is more valuable in capturing the potential heterogeneity, asymmetries, and extreme events in the impact of the pandemic on stock market volatility [15,87]. Quantile regression accounts for correcting endogenous repressors' biases [84] and gaining a deeper understanding of the effect of COVID-19 on market volatility. It mitigates sensitivity to outliers and non-Gaussian error distribution problems in panel data [89]. Following Ref. [[89], [90], [91]], the more comprehensive quantile regression for Eq. (2) and Eq. (3) is presented in Equation (4):

(4) $$Q_{y_{it}}\left(\tau |x_{it}\right)=\gamma \left(\tau \right)+x_{it}^{T}\beta_{\tau }\;\;\;\;,\;\;\;\;\;\;\;\;\;i=1,....q\;\;\;\;\;\;\;\;\;and\;\;\;\;\;\;\;\;\;t=1,.........n$$

where $Q_{y_{it}}\left(\tau |x_{it}\right)$ represents the τ conditional quantiles for $y_{it}$ (Market volatility) and $x_{it}$ denotes all the repressors (COVID-19 and Control variables) that bring change in $y_{it}$ (Market volatility). γ(τ) denotes the unobserved effect of Eq. (4). τ is the quantile value $\tau \in \left(0\;,\;1\right)$. $\beta_{\tau }$ is calculated using Equation (5).

(5) $$\beta_{\tau }=\min_{\left(\gamma ,\beta \right)}\sum_{k=1}^{m}\sum_{t=1}^{n}\sum_{i=1}^{q}\omega_{k}\rho_{\tau_{k}}\left(y_{it}-x_{it}^{T}\beta_{\tau }-\gamma \left(\tau_{k}\right)\right)+\eta \sum_{i=1}^{q}\left|\gamma_{i}\right|$$

The check function $\beta_{\tau }$ is represented by $\rho_{\tau }\left(\lambda \right)=\lambda \left(\tau -I\left(\lambda <0\right)\right)$, where the indicator function I (.) is $\lambda =y_{it}-x_{it}^{T}\beta_{\tau }-\gamma \left(\tau_{k}\right)$.

#### 3.4.2 Panel-estimated generalized least square (Panel-EGLS)

Panel-EGLS is a generalized least square procedure that can combine the features of random effect and fixed effect panel data models and account for the heterogeneity and autocorrelation in the panel data. Ref. [55,92,93] recommended that Panel-EGLS is the most suitable procedure in such a situation. It provides consistent and efficient estimates of autocorrelation and heteroscedasticity [94]. The general model of Panel-EGLS is presented in Equation (6):

(6) Y=Xφ+ε

where Y is the n×1 vector of the dependent variable (Market volatility), X is the n×p matrix of regressors (COVID-19 and Control variables) and φ is the unknown parameter. This method assumed the covariance matrix ((Ω) whose unbiased estimator can be obtained by Equation (7):

(7) $$\hat{\Omega }={\left(N-1\right)}^{-1}\sum_{i=1}^{N}\left(y_{it}-\bar{y}\right){\left(y_{it}-\bar{y}\right)}^{T}$$

Hence, the GLS estimates for φ are obtained through Equation (8):

(8) $$\varphi_{BGLS}={\left(X^{\prime }\Omega^{-1}X\right)}^{-1}X^{\prime }\Omega^{-1}Y$$

where we have assumed that $X^{\prime }\Omega^{-1}X$ is nonsingular.

## 4 Results and discussion

**Fig. 1:**
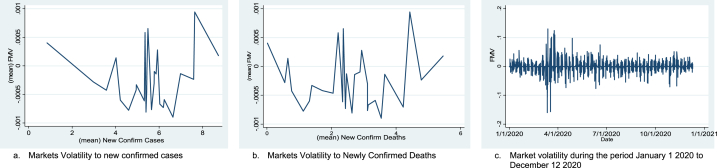
(a) Market volatility to new confirmed COVID-19 cases, (b) Market Volatility to deaths, and (c) overall Market volatility during the study period.

**Fig. 2:**
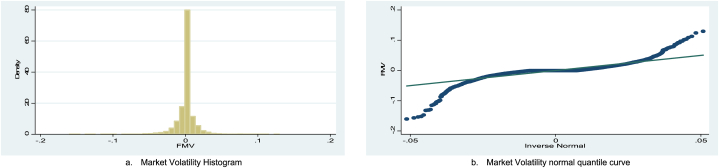
(a) The Market Volatility Histogram, and (b) Market Volatility normal quantile curve.

**Fig. 3:**
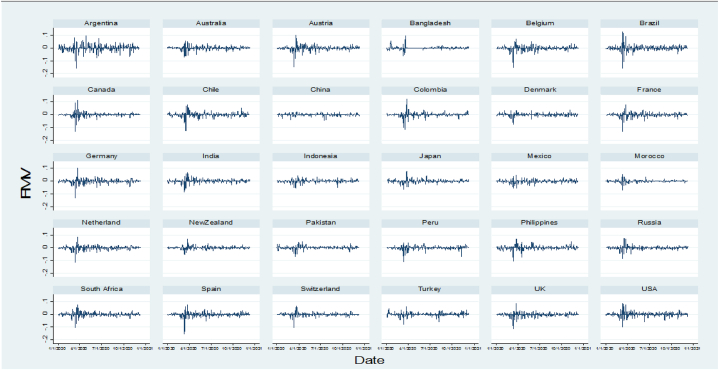
COVID-19 most affected sampled Country-wise financial market volatility from January 1, 2020, to December 12, 2020.

The overall and countrywide data behaviors of each series are presented in Fig. 1(a–c), Fig. 2(a and b), and Fig. 3. In panel data analysis, it is essential to know whether the series is stationary or non-stationary (containing unit root) [95] and to overcome spurious regression biases. Ref. [96] argued that it is essential to verify that none of the variables are integrated into order 2 (I (2)). For this purpose, different panel unit root tests (Levin, Lin & Chu, KPSS-stat, ADF, and ADF-GLS) were employed, and the results are reported in Table 3. The result of all the unit root tests indicates that all dependent, independent, and control variables are stationary at a 1 % significance level and have no unit root problem. Moreover, Table 3 also reports Variance Inflation Factor (VIF) tests. The results of the VIF test indicate that there is no high correlation in our dataset. The selection criteria of VIF is often less than 10 [97]. It means there is no high correlation when VIF is less than 10. It indicates that our data is free from multicollinearity problems.

**Table 3:** Panel data stationarity (at level) and Variance Inflation Factor (VIF) tests.

Variables	Panel unit root test				VIF
	Levin, Lin & Chu t*	KPSS	ADF	ADF-GLS	
FMV	−120.22***	82.89***	3061.74***	3094.69***	–
LnNC	−7.93***	165.79***	448.58***	335.13***	3.93
LnND	−13.81***	165.79***	457.66***	354.34***	5.75
STindex	−3.09***	164.30***	58.08**	62.37***	1.45
HDindex	−4.03***	151.63***	44.32***	44.73***	6.07
LEindex	−2.31**	161.36***	168.77***	43.27***	4.70
EPindex	−2.61***	163.41***	60.72***	27.24***	3.16

Note: '***' and '**' denote significance at 1 % and 5 % respectively.

Table 4 illustrates the results of Eq. (1) and Eq. (2). Accordingly, from Panel EGLS estimation, the coefficients of new COVID-19 cases and deaths in both the Naive and control models significantly increase financial market volatility. Table 4 also shows the quantile regression results of all the variables. The panel quantile regressions model allows us to report varied distribution patterns and is the most appropriate approach to investigate the nexus between COVID-19 and volatility.^1^ From the results, the coefficients of COVID-19 new cases at the 25th, 50th^,^ and 75th quantiles are positive. Still, at the same time, the new COVID-19 deaths are positive at the 50th and 75th quantiles, which means there is a significantly positive effect of COVID-19 deaths on volatility at the 50th and 75th quantiles. At the 25th quantile, the coefficient of new death is negative, indicating that financial market volatility is moving in the opposite direction, with COVID-19 new deaths at a lower quantile.

**Table 4:** Effect of COVID-19 on market volatility.

Depended Variable: Markets Volatility								
Variables	Panel-EGLS		Panel Quantile regressions					
	Naive Model	Control	Naive Model			Control		
			25th	50th	75th	25th	50th	75th
Constant	0.000 (0.03)	−0.012 (-1.65)*	−0.001 (−6.67)***	−0.000 (-1.14)	0.001 (0.80)	−0.002 (−0.83)	−0.000 (−2.37)**	−0.040 (-2.32)**
FMV(t-1)	−0.016 (−1.17)	−0.001 (-0.12)	−0.009 (−2.38)**	−0.000 (-18.92)***	−0.003 (-0.19)	−0.008 (-1.66)*	−0.000 (-12.25)***	−0.017 (−1.37)
LnNC(t-1)	0.000 (−2.63)***	0.000 (−2.00)**	0.000 (−2.54)**	0.000 (20.55)***	0.000 (−1.42)	0.000 (-1.87)*	0.000 (2.49)**	0.000 (−1.18)
LnND(t-1)	0.000 (1.17)	0.000 (0.62)	0.000 (2.78)***	0.000 (-15.53)***	0.000 (2.03)**	−0.000 (1.49)	0.000 (4.08)***	0.000 (2.12)**
STindex		0.000 (1.75)*				0.000 (2.77)***	−0.000 (−0.89)	0.000 (2.30)**
HDindex		−0.061 (-2.09)**				0.003 (1.29)	−0.000 (−1.22)	−0.043 (−2.87)***
LEindex		0.000 (1.25)				−0.000 (-0.22)	−0.000 (−2.79)***	0.000 (1.77)*
EPindex		0.000 (1.24)				−0.000 (-1.43)	−0.000 (-3.44)***	0.000 (1.93)**
No. of Obs.	5809	4521	5809	5809	5809	4521	4521	4521
Country effect	Yes	Yes	Yes	Yes	Yes	Yes	Yes	Yes
F-statistic	3.23**	2.29***						
Quasi-LR statistic			30.62***	68.51***	32.46***	67.23***	47.38***	43.71***

Note: ***, **, and * denote significance at Probability p < 0.01, p < 0.05, and p < 0.1, respectively. The reported values in parenthesis are t-statistics.

The positive results of new cases and deaths at naïve and controlled models in both Panel-EGLS and Quantile regression indicate that any rise in new COVID-19 deaths and cases caused the financial market to become more volatile. These results confirm our hypotheses Hb and Hc. Our results are also consistent with the prior studies, which concluded that COVID-19 positively caused financial market volatility (see, e.g., Refs. [9,24,78]. Likewise, our results, Ref. [2,60], documented a positive effect of COVID-19 on market volatility. During the COVID-19 pandemic, market risk has increased globally. The high uncertainty of the COVID-19 pandemic and its related economic losses caused the financial market to become more volatile and unpredictable [9]. However, findings reveal that the market returns are significant and negatively influenced by COVID-19, leading to volatility increase and return decrease [59]. Moreover, our results also support the behavioural view of investors because due to the rise in COVID-19 new cases and deaths, the level of uncertainty, panic, and fear is heightened, which leads to irrational behaviour of investors and increased stock market volatility [64,98]. Similarly, the COVID-19 pandemic created fear among investors, bringing market plunges and volatility spikes in financial markets [66].

At the lower 25th quantile, the influence of COVID-19 on volatility is negative and statistically significant at 1 % and 5 % levels. This heterogeneity, in effect, is evident in lower quantiles, indicating a significant influence of COVID-19 on financial volatility. The heterogeneity in the results at lower quantiles is consistent with the views that risk sensitivity to COVID-19 pandemic events differs across quantiles [66].

Table 5 illustrates the outcomes of Eq. (2) and Eq. (3) for the subsamples using panel-EGLS and quantile regression framework. In Panel A, the new COVID-19 cases positively affect the volatility in developed markets. The consequences of COVID-19 on markets are primarily addressed in the developed markets [79]. In March 2020, the Dow Jones and S&P showed that the share value dropped by 20 %, the Colombo Stock Exchange reduced by 9 %, and the Tokyo Stock Exchange share prices of the companies also fell in mid-March 2020 [49]. The stock market of Asia has become more closely linked to the financial crises of 2020 [2]. Besides the U.S. market, Asia and Europe markets also plunged. In March 2020, the U.K.'s FTSE index declined by more than 10 % [9]. In December 2019, the Japanese market dropped by more than 20 %. Panel A also reports panel quantile regression results for developed markets. Our findings report a positively significant relationship between new COVID-19 cases and death with financial volatility in developed markets at the 50th and 75th quantiles. We concluded that any rise in new COVID-19 cases and deaths caused a positive influence on financial market volatility in developed countries. Our results support our hypotheses Hb and Hc. Also, our results are consistent with the argument that potential economic losses and uncertainty from COVID-19 caused financial market volatility in developed markets [24,66,68].

**Table 5:** Effect of COVID-19 on markets volatility: Developed and emerging markets.

Panel A: Developed Markets									Panel B: Emerging Markets							
Depended Variable: Markets Volatility									Depended Variable: Markets Volatility							
Variables	Panel-EGLS		Panel Quantile regressions						Panel-EGLS		Panel Quantile regressions					
	Naive Model	Control	Naive Model			Control			Naive Model	Control	Naive Model			Control		
			25th	50th	75th	25th	50th	75th			25th	50th	75th	25th	50th	75th
Constant	−0.000 (-1.00)	−0.016 (−0.18)	−0.000 (−0.25)	−0.000 (−1.19)	0.000 (1.64)	−0.055 (-0.62)	−0.000 (-5.14)***	−0.008 (−0.06)	−0.000 (−0.25)	−0.01 (-1.14)	0.001 (0.69)	−0.000 (−3.99)***	0.001 (0.32)	−0.004 (−1.31)	−0.000 (−2.30)**	−0.025 (-1.17)
FMV(t-1)	−0.089 (−4.56)***	−0.080 (−3.32)***	−0.051 (-5.59)***	−0.000 (−18.18)	−0.036 (−2.33)**	−0.054 (−3.77)***	−0.000 (−8.67)***	−0.079 (−5.18)***	0.025 (1.39)	0.032 (1.69)*	0.048 (2.79)***	−0.000 (-3.08)***	0.048 (1.87)*	0.000 (0.06)	0.000 (0.74)	0.041 (1.52)
LnNC(t-1)	0.000 (-2.34)**	0.000 (-0.25)	−0.000 (-2.08)**	0.000 (0.87)	0.000 (-1.90)*	−0.000 (−2.37)**	0.000 (−1.73)*	0.000 (1.74)*	0.000 (−2.19)**	0.000 (-1.96)**	−0.001 (−3.14)***	0.000 (-5.70)***	0.001 (−2.15)**	−0.000 (0.34)	0.000 (-5.58)***	0.001 (-1.84)*
LnND(t-1)	0.000 (0.86)	0.000 (0.75)	−0.000 (1.64)	0.000 (7.71)***	0.000 (1.82)*	−0.000 (-0.25)	0.000 (-1.63)	0.000 (1.71)*	0.000 (1.83)*	0.000 (1.12)	−0.001 (3.88)***	0.000 (0.77)	0.001 (3.64)***	−0.000 (−0.38)	0.000 (0.90)	0.001 (2.20)**
STindex		0.000 (1.93)*				0.000 (3.27)***	0.000 (6.46)***	0.000 (2.71)***		0.000 (1.93)*				0.000 (0.71)	−0.000 (−0.93)	0.000 (1.57)
HDindex		−0.006 (−0.07)				−0.064 (−1.00)	0.000 (5.87)***	0.042 (0.51)		−0.009 (−1.04)				0.002 (1.05)	0.000 (4.56)***	−0.011 (−0.67)
LEindex		0.000 (0.13)				−0.000 (-0.78)	−0.000 (−8.62)***	0.001 (0.76)		−0.000 (−0.31)				0.000 (0.96)	−0.000 (−7.48)***	0.000 (0.14)
EPindex		0.000 (0.14)				−0.004 (−1.34)	0.000 (4.12)***	0.003 (1.07)		0.000 (0.54)				−0.000 (−1.27)	−0.000 (-4.37)***	0.000 (0.24)
No. of Obs.	2850	1785	2850	2850	2850	1785	1785	1785	2960	2728	2960	2960	2960	2728	2728	2728
Country effect	Yes	Yes	Yes	Yes	Yes	Yes	Yes	Yes	Yes	Yes	Yes	Yes	Yes	Yes	Yes	Yes
F-statistic	4.38***	3.95**							2.55***	2.41***						
Quasi-LR stat			55.79***	27.7***	42.51***	87.87***	121.50***	102.64***			111.50***	119.34***	23.76***	25.04**	74.37***	43.30***

Note: ***, **, and * represents p-value <1 %, <5 % and <10 % respectively. Parentheses reports t-statistics.

Moreover, Panel B of Table 5 illustrates the results of Eq. (2) and Eq. (3) for emerging markets. According to Panel-EGLS estimation, the effect of new COVID-19 cases and deaths on volatility in emerging markets is positive and significant at 1 % and 5 % levels, indicating that COVID-19 caused market volatility in emerging markets. The coefficient of new cases and deaths at the 50th and 75th quantiles are positive at a 1 % significant level in emerging markets while negative at the 25th quantile. Our results support our hypotheses Hb and Hc. From the results, we concluded that the COVID-19 pandemic has a similar impact on emerging markets as on developed markets.

Our study explored heterogeneity in the nexus between COVID-19 and volatility in both developed and emerging markets by differential effects on the 25th quantile against the 50th and 75th quantiles. Overall, our findings align with the view of Ref. [36,37], who reported that one country returns shocks transmitted to other countries due to connectedness. Ref. [24] found that the new COVID-19 cases and fatality rate positively influence the U.S. market volatility. Using different robust least square estimates, he also concluded a significant positive association between COVID-19 and volatility. Hence, market volatility is positively related to COVID-19 deaths, and markets become more volatile with the increase in deaths; thus, COVID-19 has emerged as a bane for market volatility and unexpected uncertainty [57].

Likewise, Ref. [80] explored the U.S. market reactions to COVID-19 and identified that COVID-19 significantly impacted volatility. Further, they argued that government restrictions on economic and commercial activities due to COVID-19 cases and deaths increased volatility [80]. At the industry level, Ref. [99] identified the variation from smaller to larger volatility and explored that economic indicators influence volatility and are sensitive to COVID-19 news. Negative news of the pandemic caused negativity biases and a significant rise in total risk at the industry level [99]. Ref. [13] examined the Asia-Pacific region's stock market volatilities and government measures. They found that government protection measures reduce market volatility in most sampled countries, such as Malaysia, Vietnam, and Laos, which faced medium volatility. At the same time, Japan, China, the Philippines, and South Korea experienced high volatility.

## 5 Conclusion and implications

The study documented the empirical nexus between the COVID-19 pandemic and market volatility of the global economy by including 30 highly affected countries due to the COVID-19 pandemic during the period ranging from January 1 to December 12, 2020. We employed quantile regression and panel-EGLS frameworks to analyze the influence of COVID-19 on volatility in the whole sample and subsamples of emerging and developed markets. Our results of panel-EGLS showed that new cases and deaths positively impact volatility. The results from quantile regression also illustrated that new deaths and cases have positively influenced market volatility at the 50th and 75th quantiles. From the subsamples, our results demonstrate almost similar signs and significance for the impact of COVID-19 on market volatility in developed and emerging markets in both the naïve and control models. From both results, we conclude that any increase in COVID-19 positively caused volatility in emerging and developed markets at the mean and upper quantile levels. Market volatility is positively related to new COVID-19 cases and deaths; markets become volatile as new cases and deaths increase. COVID-19 has appeared as a bane for market volatility and unexpected uncertainty.

The outcomes of this study carry significant implications for investors, policymakers, and governments. Our results could help investors understand market behaviour in crises, make informed decisions based on their trading strategies, and plan for future pandemics. Accordingly, our results are helpful for portfolio managers to deal with active investments in the markets, to avoid market shocks due to COVID-19, and to gain an effective optimal portfolio. Our study findings are valuable input for regulators and policymakers, who should consider the COVID-19 pandemic and any change to volatility levels in financial markets during stress periods when quantifying systematic risk. Policymakers should cover financial anomalies created by the COVID-19 pandemic to stabilize the economy. Global communities and governments should work collectively to implement mutually reinforcing actions and convey accurate information about COVID-19 in emerging and developed markets. Through this, governments will make the population calm and anxious, control measures, and make informed choices.

Additionally, all countries may implement effective public health policies to cope with the potential risk of the COVID-19 health crisis and its effect on markets. Further, our study offers some recommendations regarding COVID-19 that will be an essential consideration for governments and policymakers in future public health crises and market volatility to avoid future crises. The positive effect of new deaths and cases on volatility suggests that governments should adopt proactive measures to preserve the markets from unfavourable turmoil in future public health crises, including robust testing and contact tracing systems, vaccination efforts, and prioritizing support for vulnerable communities. Governments should adopt a monetary policy that prevents severe recessionary effects, enhances quick recovery from economic shocks, and stabilizes the volatility induced by the pandemic. This research also recommends the execution of health and economic policies during the pandemic to lessen the virus's rise and stabilize the economies.

## 6 Limitations and future directions

Covering every aspect of market volatility during the COVID-19 pandemic is challenging, so our study is limited to a certain level. First, future studies should consider high-frequency data. Second, the study included 30 countries using secondary data and analyzed the influence of COVID-19 on these countries' markets during the 1st wave of COVID-19. Therefore, future research can do this separately for each country through survey methods as the pandemic hit almost every part of the world, reaching all economic areas; it will be better to consider different robust data techniques. Third, our study did not consider the sampled countries' geographical, social, and economic contexts. Therefore, future studies may compare countries' differences and similarities based on geographical, demographic, social, and economic contexts to get more insight. Fourth, the study only uses market volatility; future studies may consider different market proxies caused by COVID-19. Fifth, the study is limited to the first wave of COVID-19; future studies could use the same procedure as ours for other waves of the COVID-19 outbreak and compare the results among different waves. Sixth, there is a need for theory development in this area, which can be possible through inductive studies; future study is needed for solid theoretical support. Lastly, most countries have recovered from the COVID-19 pandemic, but full recovery may not take place before the year 2025; future work should be designed to compare COVID-19 with the world's worst economic crises (great depression, 2008 crises) to check the effect of COVID-19 with other crises.

## Data availability

Data used for the analysis in this study are available on Public databases such as Johns Hopkins University, the Center for Systems Science and Engineering (CSSE), Global Change Data Labs (GCDL) and the global financial website (www.investing.com).

## Funding

The authors received no grant from any source.

## CRediT authorship contribution statement

**Sabeeh Ullah:** Conceptualization, Formal analysis, Methodology, Supervision, Writing – original draft, Visualization. **Sumaira Khan:** Data curation, Investigation, Validation, Software. **Nazia Iqbal Hashmi:** Resources, Validation, Writing – review & editing. **Md Shabbir Alam:** Formal analysis, Validation, Writing – review & editing.

## Declaration of competing interest

The authors declare that they have no known competing financial interests or personal relationships that could have appeared to influence the work reported in this paper.

## Figures and Tables

**Table:** 

S.No.	Country	Status	Exchange	Region	S. No.	Country	Status	Exchange	Region
1	USA	Developed	NASDAQ Composite	Americas	16	India	Emerging	BSE SENSEX	Asia & Pacific
2	France	Developed	CAC-40	Europe	17	Brazil	Emerging	BOVESPA	America
3	Denmark	Developed	OMX C20	Europe	18	Russia	Emerging	MOEX	Europe
4	U·K.	Developed	FTSE 100	Europe	19	Argentina	Emerging	SP MERVAL	America
5	Spain	Developed	IBEX 35	Europe	20	Colombia	Emerging	COLCAP	America
6	Germany	Developed	DAX	Europe	21	Mexico	Emerging	SP BMV IPC	America
7	Belgium	Developed	BEL 20	Europe	22	Bangladesh	Emerging	DSX 30	Asia & Pacific
8	Netherlands	Developed	AEX	Europe	23	Peru	Emerging	SP LIMA	America
9	Canada	Developed	SP TSX	America	24	Turkey	Emerging	BIST 100	Europe
10	Austria	Developed	ATX	Europe	25	South Africa	Emerging	SOUTH AFRICA 20	Africa
11	Switzerland	Developed	SMI	Europe	26	Indonesia	Emerging	JAKARTA COMPOSITE	Asia & Pacific
12	Japan	Developed	NEKKEI 225	Asia & Pacific	27	Chile	Emerging	S&P CLX	America
13	Australia	Developed	SP ASX 200	Asia & Pacific	28	China	Emerging	Shanghai Composite	Asia & Pacific
14	New Zealand	Developed	NZX 50	Asia & Pacific	29	Philippines	Emerging	PSEI COMPOSITE	Asia & Pacific
15	Morocco	Emerging	MORROCAN ALL	Africa	30	Pakistan	Emerging	PSX 100	Asia & Pacific

## Footnotes

1 These distribution pattern were divided into three quantiles namely lower quantile (25th), middle quantile (50th) and upper quantile (75th).
